# A Southern Dental Association

**Published:** 1868-11

**Authors:** 


*A Southern Dental Association.*—The following letter from a distinguished member of our profession, residing in Mississippi, we present to our readers: "In your last Journal I notice an editorial which fully meets my views, that is in relation to the establishment of a "Southern Dental Association," as the American Association is too large, and is made up chiefly of Eastern and Western members, with scarcely any from the South." "I do not wish to see sectional lines drawn, but, as you stated the American Association is too large for the convenience of the members South, and too far off, let it meet where it may, to suit all." "There could be *three* divisions with propriety: one East, one West, and one South; at least three divisions are evidently indicated and needed." The members in the extreme South and Southwest cannot find time and means to go so far, owing to the present condition of the country."

"I would be in favor of at once agitating this question and urging the immediate establishment of such an association, as there are already enough State and local Societies to give a full representation at the first meeting."

"This organization will cause a great many more local and State organizations throughout the South." "I would not wish to exclude representatives from any portion of the United States, nor wish to prevent Southern dentists from joining the American Association, but would like to have as much intercourse as possible." "I believe this course would engender a laudable rivalry and ambition to excell in all the higher professional attributes, and be of great benefit to both the profession and people at large." "I will write to some of the leading dentists of the South, and urge upon them their co-operation in this great enterprise."

The above letter fully expresses our views of the subject, and we hope to hear from others, and also to be able soon to inform our readers that active steps are being taken to perfect such an organization.

